# Psychological Treatment in the Management of Pain following Musculoskeletal Injury

**DOI:** 10.26502/josm.511500191

**Published:** 2025-03-31

**Authors:** Andre Aabedi, Vera Wang, Marcel P Fraix, Devendra K. Agrawal1

**Affiliations:** Departments of Translational Research, College of Osteopathic Medicine of the Pacific, Western University of Health Sciences, Pomona, California 91766 USA

**Keywords:** Acute pain, Chronic pain, Inflammation, Musculoskeletal injury, Neuropathic pain, Opioids, Pain management, Patient-centered care, Physical therapy, Rehabilitation

## Abstract

Musculoskeletal injuries are a leading cause of pain and disability, with many patients developing chronic pain. While traditional management focuses on physical treatments, psychological interventions have emerged as a complementary approach. This study examines the role of psychological treatments in pain management after musculoskeletal injury, their efficacy, and their integration with existing treatment strategies. A review of literature, including systematic reviews and meta-analyses, was conducted to assess the effectiveness of psychological treatments such as cognitive-behavioral therapy (CBT), mindfulness-based stress reduction (MBSR), and pain neuroscience education (PNE). Studies on the impact of psychological distress on pain perception, circulating inflammatory biomarkers, and neuromuscular exercises were analyzed. Research indicates that psychological elements, particularly pain catastrophizing, anxiety, and depression, play crucial roles in determining both pain intensity and disability levels. Short-term improvements in pain intensity, functional capacity, and psychological well-being have been documented with CBT, MBSR, and PNE interventions. The integration of psychological approaches with physiotherapy demonstrates enhanced patient outcomes. Biological markers of inflammation, specifically CRP and IL-6, show potential as indicators of pain severity and treatment effectiveness. Notably, neuromuscular exercises have shown pain-reducing effects comparable to pharmaceutical interventions, though long-term efficacy data for psychological treatments remains variable. The integration of psychological interventions represents a significant advancement in musculoskeletal pain management, particularly in addressing the mental and emotional dimensions of pain experience. While current research supports their immediate benefits, additional investigation is necessary to determine long-term effectiveness and refine treatment approaches. Future research should emphasize individualized treatment protocols, technological integration, and robust longitudinal studies to maximize therapeutic outcomes.

## 1. Introduction

Musculoskeletal injuries are a significant source of pain and disability globally, with a substantial proportion of individuals experiencing persistent pain long after the initial injury. Treatment primarily relies on physical therapies such as rest, ice, heat, compression, or elevation, but psychological treatments addressing emotional and cognitive aspects of pain perception have also emerged as a possible approach [1]. The risk factors for chronic pain following musculoskeletal injury include non-modifiable factors (female sex, age over 65 years, and low socioeconomic status) and modifiable risk factors (greater pain catastrophizing, higher pain-related fear, and increased symptoms of anxiety, depression, and post-traumatic stress disorder (PTSD)) [1].

Chronic pain after musculoskeletal injury involves both physiological and psychological components. Psychological factors such as catastrophic thinking, depression, anxiety, and PTSD are strongly associated with increased pain intensity and disability [2]. These factors can influence the perception of pain and the overall recovery process [3]. The Orthopedic Trauma Association highlights that pain catastrophizing and other ineffective coping strategies are consistently linked to greater pain intensity and disability [2]. The incidence of chronic pain following musculoskeletal injury is notable. For instance, a study found that at six months post-injury, 43.9% of patients reported some degree of pain, and 10.1% experienced chronic pain [4]. Psychological interventions, including cognitive-behavioral therapy (CBT), mindfulness training, and other mindset training, have shown promise in reducing pain, functional impairment, and psychological distress in these patients [5].

Current research demonstrates conflicting evidence about the efficacy of psychological treatments. A systematic review and meta-analysis by Aaron et al. [6] included 24 randomized controlled trials with a total of 1966 participants and found that psychological interventions, such as cognitive behavioral therapy (CBT), significantly reduced pain intensity, functional impairment, and symptoms of depression, anxiety, and PTSD immediately post-treatment [6]. However, the certainty of evidence was low, and the effects on pain and functional impairment were not sustained at six-month follow-up [6].

Although psychological interventions alone are controversial, they may be particularly effective when combined with physiotherapy care. A network meta-analysis by Ho et al. [7] demonstrated that CBT and pain education, when delivered alongside physiotherapy, resulted in significant improvements in physical function and pain intensity. This integrative approach underscores the necessity of a multimodal strategy in managing chronic musculoskeletal pain. Addressing psychological distress is supported by medical organizations such as the Orthopedic Trauma Association and the American College of Physicians. The Orthopedic Trauma Association's clinical practice guidelines note that interventions like CBT, self-management training, and peer support can improve mental health outcomes and reduce pain [1]. Similarly, the American College of Physicians recommends psychological therapies, including mindfulness-based stress reduction and progressive relaxation therapy, for chronic low back pain, emphasizing their role in improving pain intensity and functional status [8].

Despite promising results, there is a need for further research to identify the most effective psychological treatments and to understand their long-term benefits. The American Society of Anesthesiologists and the American Society of Regional Anesthesia and Pain Medicine recommend incorporating psychological treatments such as CBT, biofeedback, and relaxation training into chronic pain management protocols [9]. Thus, psychological treatments offer a valuable adjunct to traditional pain management strategies for musculoskeletal injuries. While current evidence supports their efficacy in reducing pain and psychological distress, ongoing research is essential to optimize these interventions and ensure sustained benefits for patients.

## 2. Underlying Cellular and Molecular Mechanisms of Pain in Musculoskeletal Injuries

**Figure 1:**
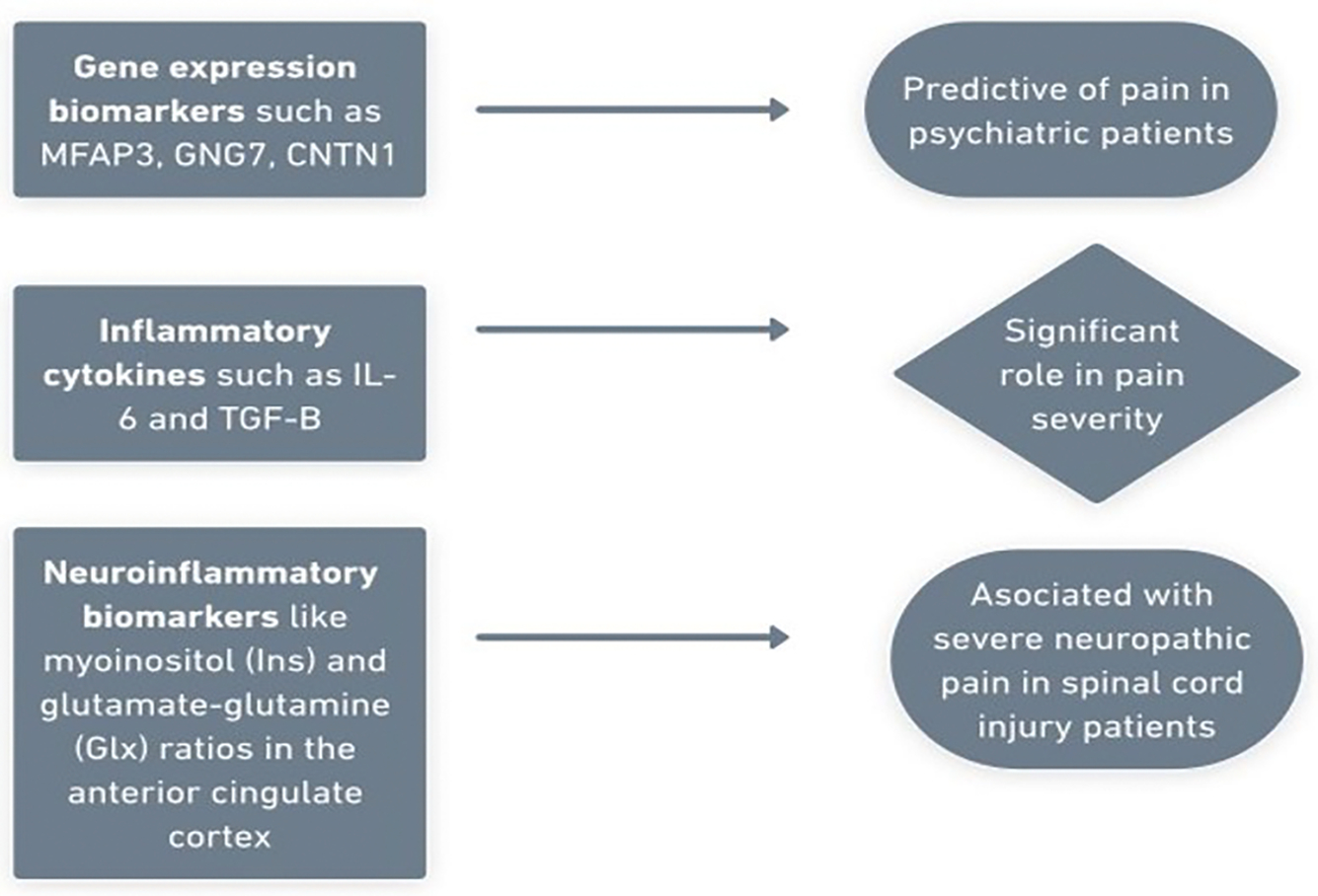
The schematic diagram illustrating the major biomarkers involved in the severity of pain include various gene expression markers, inflammatory cytokines, and neuroinflammatory mediators.

The underlying cellular and molecular mechanisms of pain in musculoskeletal injuries involve a complex interplay between peripheral and central nervous system components [10–14]. Peripheral nociceptors become sensitized following injury due to inflammation or ischemia, leading to increased pain transmission signals to the spinal cord [15–18]. This process involves the release of neurotransmitters such as substance P and calcitonin gene-related peptide (CGRP) from primary afferent neurons, which further potentiate inflammation and pain perception [10–12] (Figure 1).

In the spinal cord, dorsal horn neurons become sensitized, exhibiting increased background activity, receptive field size, and responses to stimuli. This sensitization is mediated by the activation of N-methyl-D-aspartate (NMDA) receptors, non-NMDA excitatory amino acid receptors, and neurokinin 1 receptors [15]. Central sensitization, characterized by enhanced excitability and reduced inhibition within the central nervous system, plays a crucial role in the persistence of pain [15]. Psychological factors significantly influence the perception and management of pain in musculoskeletal injuries. Pain catastrophizing, anxiety, depression, and other psychosocial factors can exacerbate pain intensity and hinder recovery. The Orthopedic Trauma Association highlights that variations in pain intensity are more closely associated with psychosocial factors than with the severity of the injury itself [19]. Addressing these psychological aspects through cognitive-behavioral therapy and other psychological interventions can be effective in managing pain and improving outcomes for patients with musculoskeletal injuries [19].

## 3. Circulating Inflammatory Biomarkers to Predict the Severity of Pain and Effect of Interventions

Circulating inflammatory biomarkers have been investigated for their potential to predict the severity of pain and the effect of interventions in patients with musculoskeletal injuries. Several studies have explored the relationship between these biomarkers and pain outcomes, particularly in the context of psychological treatments for pain management.

For instance, Tonelli et al. [20] found that biomarkers such as neuropeptide Y (NPY), E-Selectin, vitamin D, and C-reactive protein (CRP) were associated with pain levels in patients with acute or subacute low back pain. These biomarkers showed varying degrees of correlation with pain and disability outcomes following different interventions, suggesting their potential utility in predicting treatment responses [20]. Lee et al. [21] highlighted the moderating effects of psychological and social variables on the association between inflammatory biomarkers and pain severity. They found that factors such as employment status, pre-existing psychopathology, and sex could influence the relationship between biomarkers like TNF-α, TGF-β1, and CRP with pain severity and interference, indicating the complex interplay between biological and psychosocial factors in pain perception [21].

Simon et al. [22] demonstrated that higher levels of CRP, IL-6, and IL-10 were associated with greater pain reduction following exercise-induced shoulder injury in a high-risk genetic and psychological subgroup. This suggests that these biomarkers could help predict pain changes in response to physical interventions [22]. Hedderson et al. [23] also reported that elevated concentrations of IL-6 and IL-10 were associated with pain intensity following exercise-induced shoulder muscle injury, further supporting the role of inflammatory cytokines in pain modulation.

Overall, these studies suggest that circulating inflammatory biomarkers, particularly CRP, IL-6, IL-10, and TNF-α, may serve as valuable predictors of pain severity and treatment outcomes in patients with musculoskeletal injuries [15–17,19,21]. However, the influence of psychological and social factors must also be considered to fully understand and utilize these biomarkers in clinical practice.

## 4. Components and Effectiveness of Pain Neuroscience Education and Effect on Anxiety and Distress

Pain Neuroscience Education (PNE) is an intervention designed to reconceptualize an individual's understanding of their pain, aiming to reduce its perceived threat. The core components of PNE include educating patients about the neurophysiology and neurobiology of pain, emphasizing that pain is a protective mechanism rather than a direct indicator of tissue damage. This education helps patients understand that chronic pain can be influenced by various factors, including psychological and social elements.

The effectiveness of PNE in managing pain after musculoskeletal injury has been evaluated in several studies. A systematic review and meta-analysis by Watson et al. [24] found that PNE had low clinical relevance in the short term for pain and disability but showed clinically relevant effects on kinesiophobia and pain catastrophizing in the medium term [24]. Another meta-analysis by Salazar-Méndez et al. [25] highlighted that longer durations of PNE were associated with significant improvements in anxiety symptoms, catastrophizing, and kinesiophobia, suggesting a dose response relationship [25].

Furthermore, Bülow et al. [26] reported small to moderate effects of PNE on pain and psychological distress at post-intervention and long-term follow-up, although the overall quality of evidence was low [26]. Louw et al. [27] also demonstrated that PNE could reduce anxiety and stress in patients with chronic musculoskeletal pain.

## 5. Pharmacological and Non-Pharmacological Treatments in Pain Management

Pain management of musculoskeletal injury is multifactorial and depends on severity, including both pharmacological and non-pharmacological interventions. Typically, they are combined but in mild cases solely non-pharmacological treatments including rest, ice, compression, and elevation may be utilized. Pharmacological management typically includes the use of nonsteroidal anti-inflammatory drugs (NSAIDs), acetaminophen, and in some cases, opioids. The use of opioids should be approached with caution due to the risk of dependency and the potential for misdiagnosing psychological distress as physical pain [1].

Psychological interventions present as an additional method of managing pain. The Orthopedic Trauma Association highlights the importance of addressing psychological distress, which can manifest as anxiety, depression, and post-traumatic stress disorder (PTSD) following injury. Cognitive behavioral therapy (CBT), self-management interventions, educational information access, peer support, and online social networking have been studied, with CBT showing positive effects on pain relief in some trials [19].

The American College of Physicians recommends psychological therapies such as progressive relaxation, electromyography biofeedback, operant therapy, and mindfulness-based stress reduction for chronic low back pain, noting moderate improvements in pain intensity and functional status [2]. Additionally, a systematic review and meta-analysis found that psychological treatments can reduce pain intensity, functional impairment, and symptoms of depression, anxiety, and PTSD immediately post-treatment, although the certainty of evidence is low [6].

## 6. Neuromuscular Exercises and Psychological Treatment Strategies and How Do They Work, and Their Effectiveness Compared To Pharmacological Treatment?

Neuromuscular exercises and psychological treatment strategies are integral components in the management of chronic musculoskeletal pain. Neuromuscular exercises, which include activities such as motor control training and proprioceptive exercises, aim to improve muscle strength, coordination, and joint stability. These exercises can induce neuroplastic changes in the nervous system, which are beneficial for patients with chronic musculoskeletal disorders [28].

Psychological treatment strategies, such as cognitive-behavioral therapy (CBT), pain neuroscience education (PNE), and mindfulness-based stress reduction (MBSR), target the cognitive and emotional aspects of pain. These interventions help patients develop coping strategies, reduce pain catastrophizing, and improve overall mental health. The American College of Physicians recommends psychological therapies, including CBT and mindfulness-based stress reduction, as effective nonpharmacologic treatments for chronic low back pain [8].

When comparing these nonpharmacological treatments to pharmacological treatments, evidence suggests that neuromuscular exercises and psychological interventions can be as effective, if not more so, in the long term. Pharmacological treatments, such as NSAIDs and opioids, provide short-term pain relief but are associated with potential adverse effects and do not address the underlying psychological and functional aspects of chronic pain [29].

**Figure 2:**
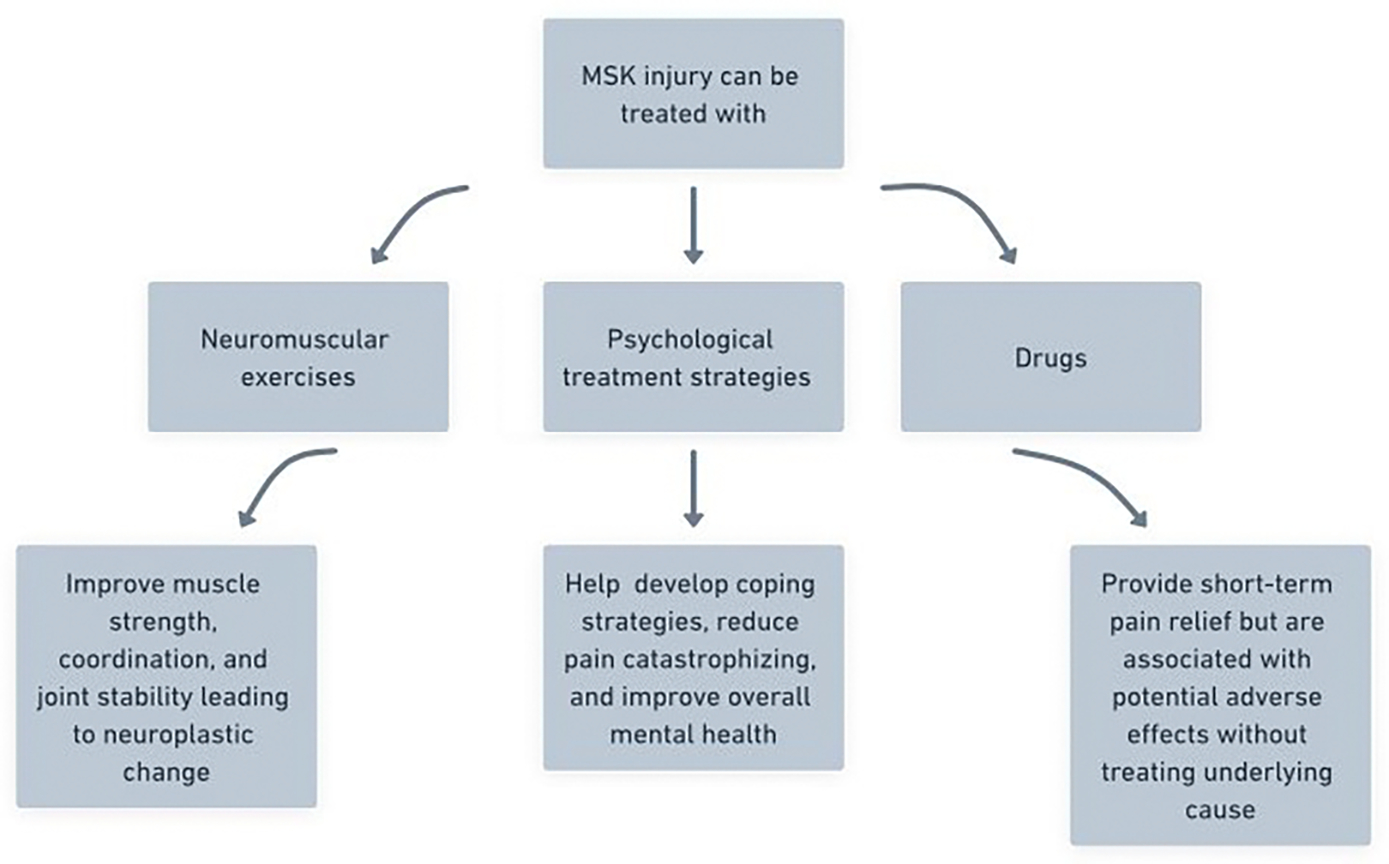
The flow chart illustrates various treatment modalities and their benefits following an MSK injury.

Psychological treatments play a crucial role in the management of pain after musculoskeletal injury. A systematic review and meta-analysis found that psychological interventions, such as CBT and mindfulness, can significantly reduce pain intensity, functional impairment, and symptoms of depression, anxiety, and PTSD immediately post-treatment [30]. These interventions are particularly valuable as they address the psychological distress and maladaptive coping strategies that often accompany chronic pain, leading to improved patient outcomes (Figure 2).

## 7. Outstanding Questions, Gaps in our knowledge and Challenges

Psychological treatments for managing pain after musculoskeletal injury have shown promise, but several gaps in knowledge remain. One major challenge is the variability in treatment and injury characteristics across studies, which complicates the synthesis of evidence. The Orthopedic Trauma Association highlights that while cognitive-behavioral therapy (CBT) and other psychosocial interventions can improve mental health and reduce symptoms of depression, anxiety, and PTSD, the evidence for their effectiveness in reducing pain and functional impairment is still uncertain due to low certainty of results [1].

Another limitation is the lack of high-quality research. Many studies included in systematic reviews are of low or very low quality, with substantial heterogeneity and risk of bias [6]. This limits the ability to draw definitive conclusions about the efficacy of psychological treatments. Additionally, there is a need for more research on the long-term effects of these interventions, as most studies focus on short-term outcomes, and the benefits observed immediately post-treatment often diminish over time [6].

Furthermore, there is a need to better understand the mechanisms through which psychological interventions exert their effects. Mediation and moderation analyses suggest that reductions in pain-related fear, catastrophizing, and increases in self-efficacy are important mediators of treatment effects, but the evidence is not robust [31]. Identifying specific treatment targets and understanding how they influence outcomes can help refine and optimize psychological interventions.

Lastly, implementation challenges persist. Physiotherapists and other healthcare providers often feel inadequately trained to deliver biopsychosocial interventions, and there are organizational barriers such as time constraints and referral pathways that hinder the integration of psychological treatments into routine care [32]. Addressing these challenges through better training, support, and systemic changes is crucial for improving the uptake and effectiveness of psychological treatments for pain management after musculoskeletal injury.

## 8. Future Direction

Future research on psychological treatment for the management of pain after musculoskeletal injury should focus on several key areas to enhance treatment efficacy and implementation. Firstly, there is a need for personalized and process-based approaches. McCracken [33] argues that the development of truly personalized treatments, which consider individual variability and contextually sensitive ongoing assessment, is crucial for improving outcomes in chronic pain management. This approach should be idiographic, focusing on evidence-based mechanisms of change tailored to individual needs. Secondly, the integration of technology-enhanced delivery models can facilitate the implementation of psychologically informed practices. Doorley et al. [34] highlight the potential of technology to overcome barriers to implementation and propose future research directions to explore these models further. This includes the use of telehealth, mobile applications, and other digital tools to deliver psychological interventions effectively. Additionally, research should investigate the specific mediators and moderators of treatment effects. Murillo et al. [35] emphasize the importance of understanding how psychologically based interventions work by examining pain-related fears, cognitions, and behaviors. Identifying these factors can help refine and target interventions more precisely. Finally, there is a need for high-quality, long-term studies to assess the durability of treatment effects. Aaron et al. [30] noted that while psychological treatments show promise in reducing pain and psychological distress post-treatment, the certainty of evidence is low, and long-term benefits are unclear. Future research should aim to conduct rigorous trials with extended follow-up periods to establish the sustained efficacy of these interventions.

In summary, advancing research in this field requires a focus on personalized, technology-enhanced, and mechanism-based approaches, alongside robust long-term studies to validate and optimize psychological treatments for pain management after musculoskeletal injury.

## 9. Conclusion

The management of pain following musculoskeletal injury indeed requires an integrative approach that considers physical, psychological, and social factors. Traditional treatments such as pharmacological interventions and physiotherapy are essential, but psychological treatments also play a significant role in addressing the emotional and cognitive dimensions of pain. Psychological treatments, including cognitive-behavioral therapy (CBT), mindfulness-based stress reduction (MBSR), and pain neuroscience education, have shown potential in reducing pain perception and mitigating psychological distress such as anxiety, depression, and pain catastrophizing. For instance, a systematic review and meta-analysis found that psychological treatments can lead to lower pain intensity, functional impairment, and symptoms of depression, anxiety, and PTSD immediately post-treatment, although the certainty of evidence was low or very low for most effects [30]. The American College of Physicians recommends psychological therapies, including CBT and MBSR, for chronic low back pain, highlighting their effectiveness in improving pain and function [8]. Similarly, the Orthopedic Trauma Association emphasizes the importance of psychosocial interventions, including CBT and self-management strategies, in managing pain and psychological distress following musculoskeletal injuries [19]. However, challenges such as variability in study designs, inconsistent evidence regarding long-term benefits, and limited understanding of underlying mechanisms necessitate more rigorous research. Implementation barriers, including inadequate training for healthcare providers and organizational constraints, also hinder the integration of psychological treatments into routine care [30].

Future directions should focus on personalized and technology-enhanced approaches to psychological treatment, leveraging telehealth and digital tools to expand accessibility. Long term, high-quality studies are essential to establish the sustained efficacy of these interventions and optimize their application in clinical practice [34].

In conclusion, psychological treatments are a critical component of pain management strategies for musculoskeletal injuries. By addressing both the physical and psychological aspects of pain, these interventions can enhance recovery, improve quality of life, and potentially prevent the transition to chronic pain. Advancing research and overcoming implementation barriers will be key to realizing the full potential of psychological therapies in pain management.

## 10. Key Points

- Effective pain management after musculoskeletal injury requires a multimodal approach that balances analgesia with minimizing risks associated with opioid use.
- Musculoskeletal injury often leads to acute pain, which, if inadequately treated, can progress to chronic pain and functional impairment.
- Opioids remain a cornerstone of pain management for severe musculoskeletal injuries, but their use must be carefully monitored to avoid dependence and adverse effects.
- Patients experiencing acute pain after injury benefit from early intervention strategies that include both pharmacologic and non-pharmacologic treatments.
- Failure to adequately manage acute pain may increase the risk of transition to chronic pain, significantly impacting long-term outcomes.
- Physical therapy is an essential component of rehabilitation, helping to restore function and prevent the development of chronic pain.
- Some patients may develop neuropathic pain, which requires alternative treatment strategies, including anticonvulsants or antidepressants.
- Early and structured rehabilitation is crucial in preventing functional decline and promoting long-term recovery.
- Patient-centered care in pain management involves tailoring treatments to individual patient needs, preferences, and risk factors.
- Targeting inflammation with NSAIDs can help reduce pain and swelling in the acute phase of musculoskeletal injuries.

## References

[1] Hsu JR, Mir H, Wally MK, Orthopaedic Trauma Association Musculoskeletal Pain Task Force. Clinical Practice Guidelines for Pain Management in Acute Musculoskeletal Injury. J Orthop Trauma 33 (2019): e158–e182. 30681429 10.1097/BOT.0000000000001430 PMC6485308

[2] Silva Guerrero AV, Maujean A, Campbell L, A Systematic Review and Meta-Analysis of the Effectiveness of Psychological Interventions Delivered by Physiotherapists on Pain, Disability and Psychological Outcomes in Musculoskeletal Pain Conditions. Clin J Pain 34 (2018): 838–857. 29554030 10.1097/AJP.0000000000000601

[3] Vranceanu AM, Bachoura A, Weening A, Psychological factors predict disability and pain intensity after skeletal trauma. J Bone Joint Surg Am 96 (2014): e20. 24500592 10.2106/JBJS.L.00479

[4] Pierik JGJ, IJzerman MJ, Gaakeer MI, Incidence and prognostic factors of chronic pain after isolated musculoskeletal extremity injury. Eur J Pain Lond Engl 20 (2016): 711–722. 10.1002/ejp.796 26492564

[5] Kang KK, Ciminero ML, Parry JA, The Psychological Effects of Musculoskeletal Trauma. J Am Acad Orthop Surg 29 (2021): e322–e329. 33475305 10.5435/JAAOS-D-20-00637

[6] Aaron RV, Rassu FS, Wegener ST, Psychological treatments for the management of pain after musculoskeletal injury: a systematic review and meta-analysis. Pain 165 (2024): 3–17. 37490624 10.1097/j.pain.0000000000002991 PMC10808265

[7] Ho EKY, Chen L, Simic M, Psychological interventions for chronic, non-specific low back pain: systematic review with network meta-analysis. BMJ 376 (2022): e067718. 35354560 10.1136/bmj-2021-067718 PMC8965745

[8] Qaseem A, Wilt TJ, McLean RM, Noninvasive Treatments for Acute, Subacute, and Chronic Low Back Pain: A Clinical Practice Guideline From the American College of Physicians. Ann Intern Med 166 (2017): 514–530. 28192789 10.7326/M16-2367

[9] American Society of Anesthesiologists Task Force on Chronic Pain Management, American Society of Regional Anesthesia and Pain Medicine. Practice guidelines for chronic pain management: an updated report by the American Society of Anesthesiologists Task Force on Chronic Pain Management and the American Society of Regional Anesthesia and Pain Medicine. Anesthesiology 112 (2010): 810–833. 20124882 10.1097/ALN.0b013e3181c43103

[10] Sluka KA. Pain mechanisms involved in musculoskeletal disorders. J Orthop Sports Phys Ther 24 (1996): 240–254. 8892139 10.2519/jospt.1996.24.4.240

[11] Kirkpatrick DR, McEntire DM, Smith TA, Transmission pathways and mediators as the basis for clinical pharmacology of pain. Expert Rev Clin Pharmacol 9 (2016): 1363–1387. 27322358 10.1080/17512433.2016.1204231 PMC5215101

[12] McEntire DM, Kirkpatrick DR, Dueck NP, Pain transduction: a pharmacologic perspective. Expert Rev Clin Pharmacol 9 (2016): 1069–80. 27137678 10.1080/17512433.2016.1183481 PMC4975548

[13] Raney EB, Thankam FG, Dilisio MF, Pain and the pathogenesis of biceps tendinopathy. Am J Transl Res 9 (2017): 2668–2683. 28670360 PMC5489872

[14] Kirkpatrick DR, McEntire DM, Hambsch ZJ, Therapeutic Basis of Clinical Pain Modulation. Clin Transl Sci 8 (2015): 848–56.. 25962969 10.1111/cts.12282 PMC4641846

[15] Basbaum AI, Bautista DM, Scherrer G, Cellular and molecular mechanisms of pain. Cell 139 (2009): 267–284. 19837031 10.1016/j.cell.2009.09.028 PMC2852643

[16] Werner JH, Rosenberg JH, Keeley KL, Immunobiology of periprosthetic inflammation and pain following ultra-high-molecular-weight-polyethylene wear debris in the lumbar spine. Expert Rev Clin Immunol 14 (2018): 695–706. 30099915 10.1080/1744666X.2018.1511428 PMC6287907

[17] Eskandar T, Ahmed Z, Pan J, The Decline of Lumbar Artificial Disc Replacement. J Spine Res Surg 6 (2024): 86–92. 39267915 10.26502/fjsrs0078 PMC11392031

[18] Rajalekshmi R, Agrawal DK. Understanding Fibrous Tissue in the Effective Healing of Rotator Cuff Injury. J Surg Res (Houst) 7 (2024): 215–228. 38872898 10.26502/jsr.10020363 PMC11174978

[19] Hsu JR, Mir H, Wally MK, Orthopaedic Trauma Association Musculoskeletal Pain Task Force. Clinical Practice Guidelines for Pain Management in Acute Musculoskeletal Injury. J Orthop Trauma 33 (2019): e158–e182. 30681429 10.1097/BOT.0000000000001430 PMC6485308

[20] Tonelli Enrico V, Schneider M, Haas M, The association of biomarkers with pain and function in acute and subacute low back pain: a secondary analysis of an RCT. BMC Musculoskelet Disord 23 (2022): 1059. 36471334 10.1186/s12891-022-06027-9 PMC9721012

[21] Lee JY, Fakhereddin M, MacDermid JC, An Exploration of Blood Marker×Environment Interaction Effects on Pain Severity and Interference Scores in People With Acute Musculoskeletal Trauma. Clin J Pain 37 (2021): 747–758. 34292185 10.1097/AJP.0000000000000961

[22] Simon CB, Bishop MD, Wallace MR, Circulating Inflammatory Biomarkers Predict Pain Change Following Exercise-Induced Shoulder Injury: Findings From the Biopsychosocial Influence on Shoulder Pain Preclinical Trial. J Pain 24 (2023): 1465–1477. 37178095 10.1016/j.jpain.2023.04.001 PMC10523877

[23] Hedderson WC, Borsa PA, Fillingim RB, Plasma Concentrations of Select Inflammatory Cytokines Predicts Pain Intensity 48 Hours Post-Shoulder Muscle Injury. Clin J Pain 36 ( 2020): 775–781. 32675582 10.1097/AJP.0000000000000861 PMC7484373

[24] Watson JA, Ryan CG, Cooper L, Pain Neuroscience Education for Adults With Chronic Musculoskeletal Pain: A Mixed-Methods Systematic Review and Meta-Analysis. J Pain 20 (2019): 1140.e1–1140.e22. 10.1016/j.jpain.2019.02.011 30831273

[25] Salazar-Méndez J, Núñez-Cortés R, Suso-Martí L, Dosage matters: Uncovering the optimal duration of pain neuroscience education to improve psychosocial variables in chronic musculoskeletal pain. A systematic review and meta-analysis with moderator analysis. Neurosci Biobehav Rev 153 (2023): 105328. 37516218 10.1016/j.neubiorev.2023.105328

[26] Bülow K, Lindberg K, Vaegter HB, Effectiveness of Pain Neurophysiology Education on Musculoskeletal Pain: A Systematic Review and Meta-Analysis. Pain Med Malden Mass 22 (2021): 891–904. 10.1093/pm/pnaa484 33764394

[27] Louw A, Diener I, Butler DS, The effect of neuroscience education on pain, disability, anxiety, and stress in chronic musculoskeletal pain. Arch Phys Med Rehabil 92 (2011): 2041–2056. 22133255 10.1016/j.apmr.2011.07.198

[28] Pelletier R, Higgins J, Bourbonnais D. Addressing Neuroplastic Changes in Distributed Areas of the Nervous System Associated With Chronic Musculoskeletal Disorders. Phys Ther 95 (2015): 15821591. 10.2522/ptj.20140575 25953594

[29] Babatunde OO, Jordan JL, Van der Windt DA, Effective treatment options for musculoskeletal pain in primary care: A systematic overview of current evidence. PloS One 12 (2017): e0178621. 28640822 10.1371/journal.pone.0178621 PMC5480856

[30] Aaron RV, Rassu FS, Wegener ST, Psychological treatments for the management of pain after musculoskeletal injury: a systematic review and meta-analysis. Pain 165 (2024): 3–17. 37490624 10.1097/j.pain.0000000000002991 PMC10808265

[31] Murillo C, Vo TT, Vansteelandt S, How do psychologically based interventions for chronic musculoskeletal pain work? A systematic review and meta-analysis of specific moderators and mediators of treatment. Clin Psychol Rev 94 (2022): 102160. 35561510 10.1016/j.cpr.2022.102160 PMC11146991

[32] Holopainen R, Simpson P, Piirainen A, Physiotherapists’ perceptions of learning and implementing a biopsychosocial intervention to treat musculoskeletal pain conditions: a systematic review and metasynthesis of qualitative studies. Pain 161 (2020): 1150–1168. 31977935 10.1097/j.pain.0000000000001809

[33] McCracken LM. Personalized pain management: Is it time for process-based therapy for particular people with chronic pain? Eur J Pain Lond Engl 27 (2023): 1044–1055. 10.1002/ejp.2091 36755478

[34] Doorley JD, Lentz TA, Yeh GY, Technology-Enhanced Delivery Models to Facilitate the Implementation of Psychologically Informed Practice for Chronic Musculoskeletal Pain Phys Ther 103 (2022): pzac141. 36210757 10.1093/ptj/pzac141 PMC10071498

[35] Murillo C, Vo TT, Vansteelandt S, How do psychologically based interventions for chronic musculoskeletal pain work? A systematic review and meta-analysis of specific moderators and mediators of treatment. Clin Psychol Rev 94 (2022): 102160. 35561510 10.1016/j.cpr.2022.102160 PMC11146991

